# Supplementary figures and images for: SNORD90 induces glutamatergic signaling following treatment with monoaminergic antidepressants
Source: eLife. 2023 Jul 11;12:e85316. doi: 10.7554/eLife.85316 (PMC10335830; doi:10.7554/eLife.85316)

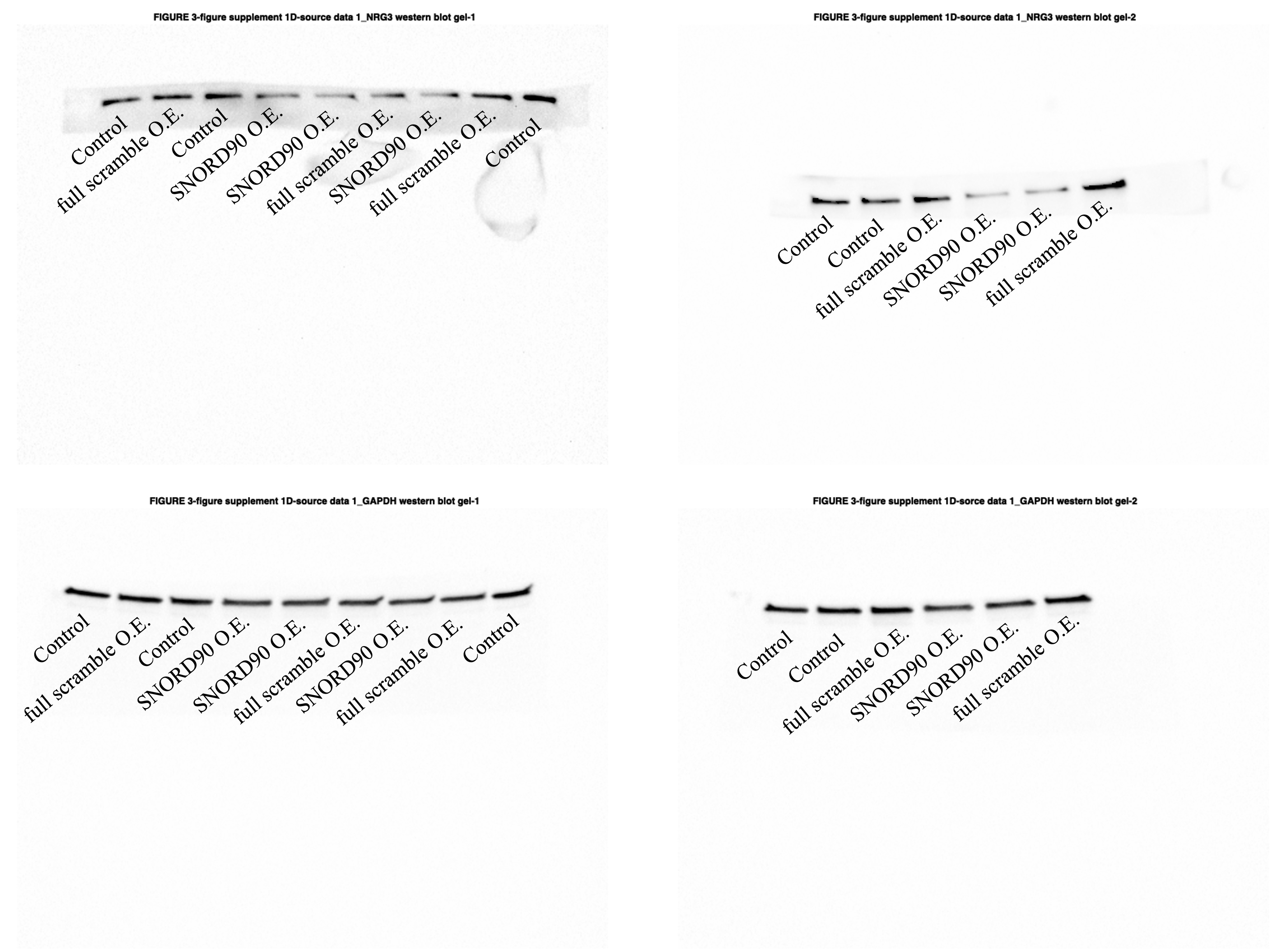

Supplement: Figure 3—figure supplement 1—source data 1. [file elife-85316-fig3-figsupp1-data1.zip › Figure 3-figure supplement 1-source data 1.tiff]

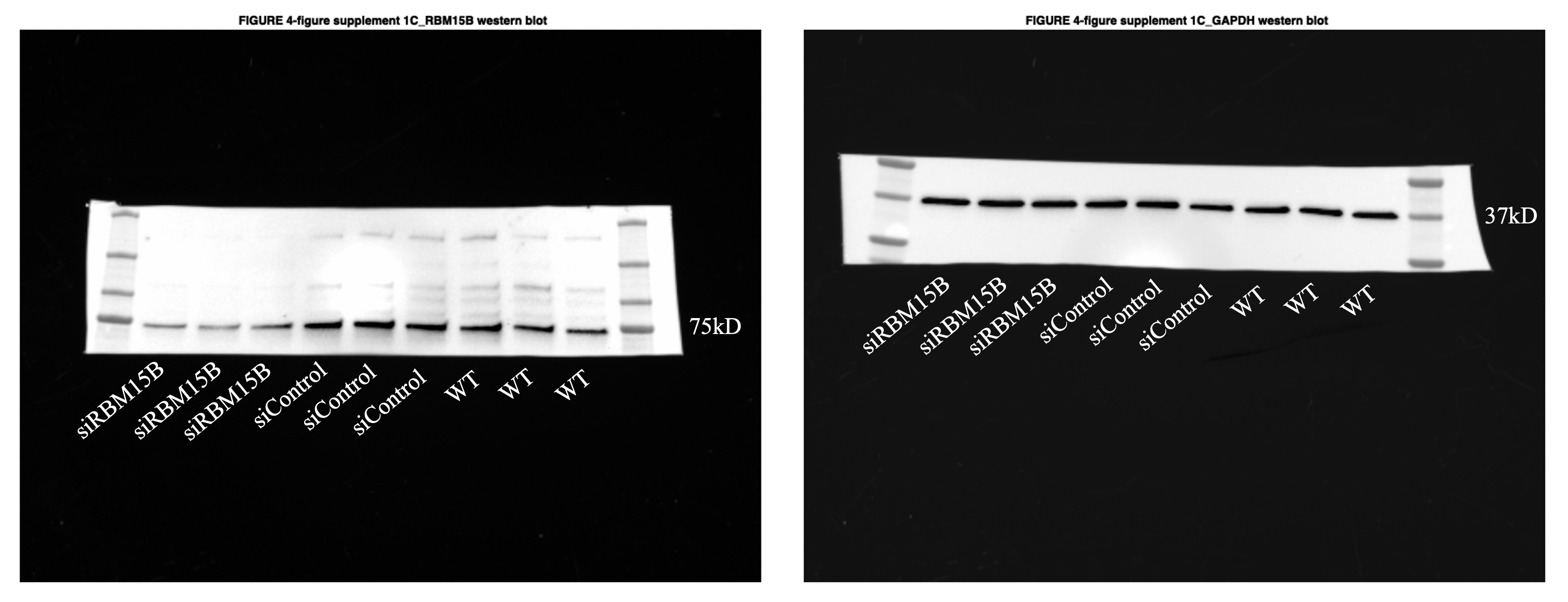

Supplement: Figure 4—figure supplement 1—source data 1. [file elife-85316-fig4-figsupp1-data1.zip › Figure 4-figure supplement 1-source data 1.tiff]

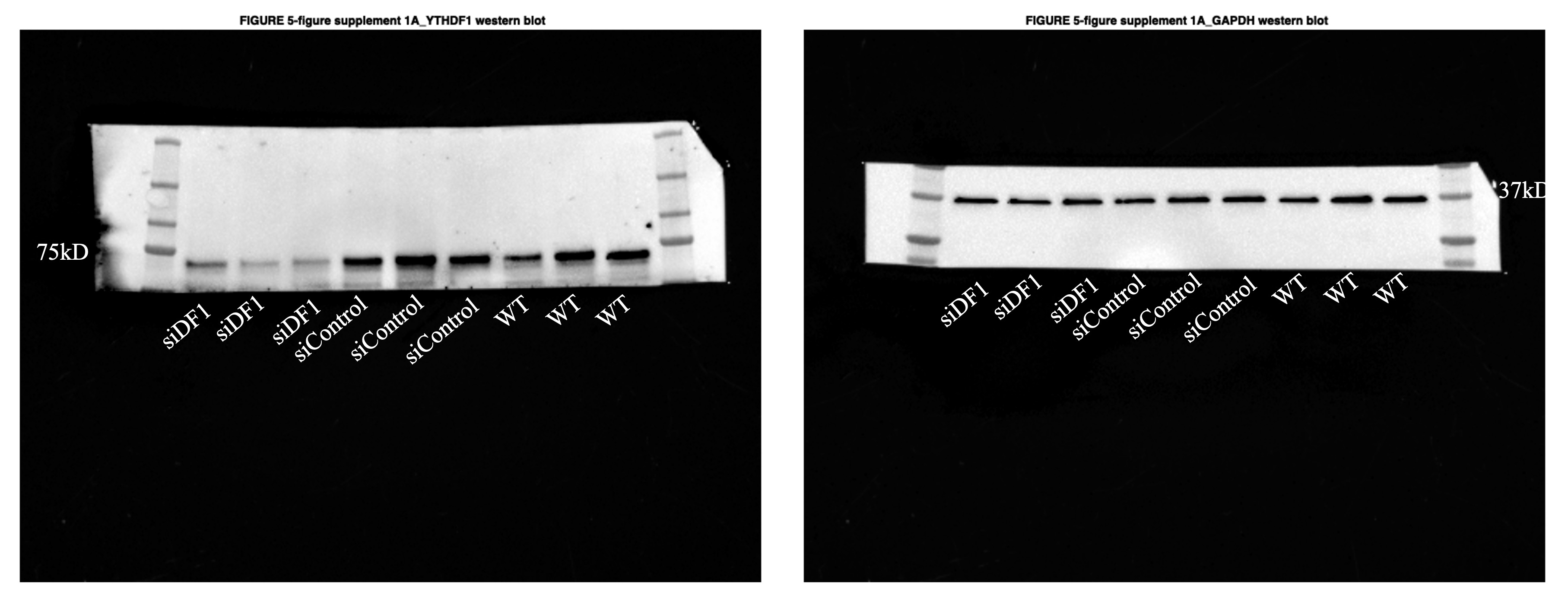

Supplement: Figure 5—figure supplement 1—source data 1. [file elife-85316-fig5-figsupp1-data1.zip › Figure 5-figure supplement 1-source data 1.tiff]

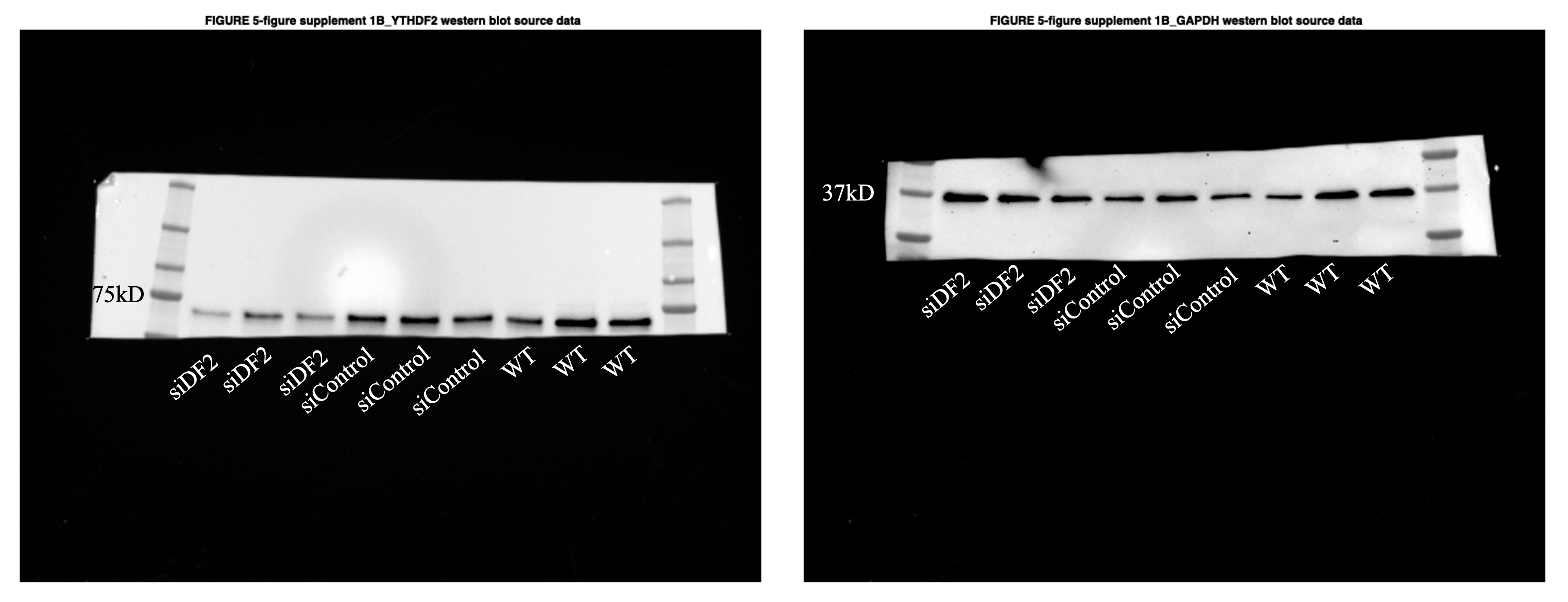

Supplement: Figure 5—figure supplement 1—source data 2. [file elife-85316-fig5-figsupp1-data2.zip › Figure 5-figure supplement 1-source data 2.tiff]

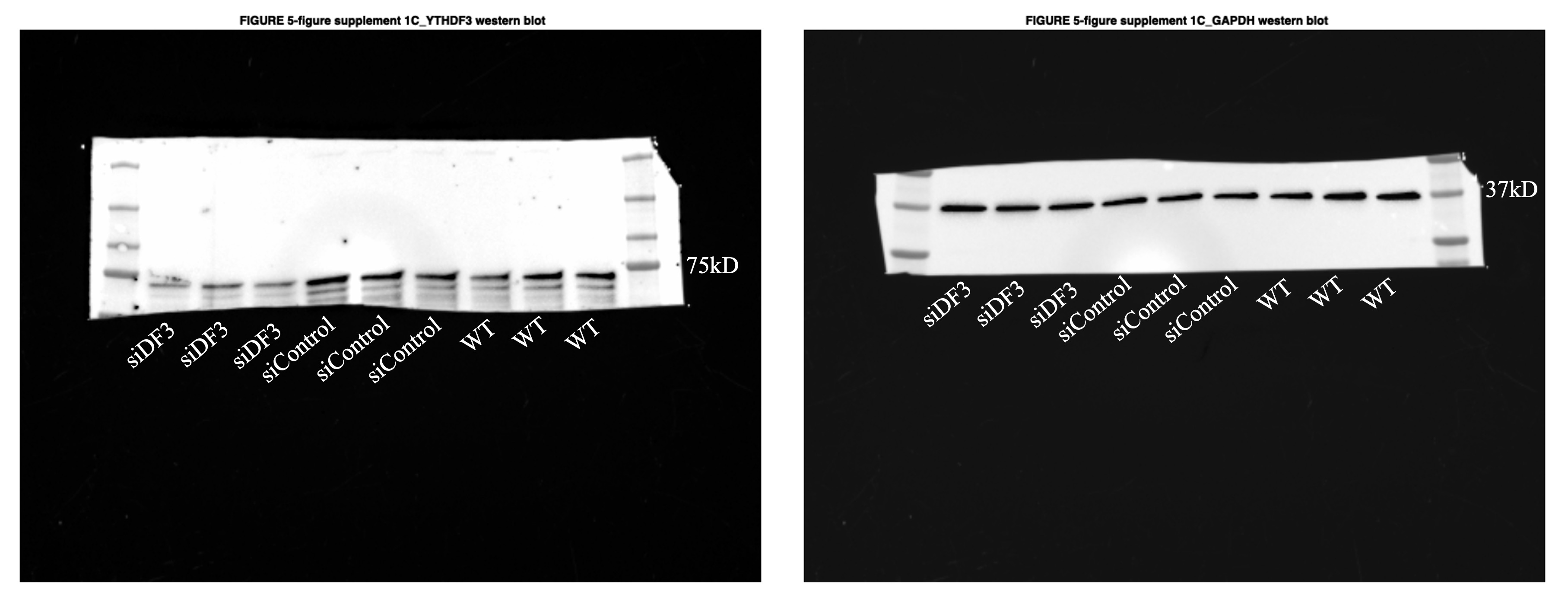

Supplement: Figure 5—figure supplement 1—source data 3. [file elife-85316-fig5-figsupp1-data3.zip › Figure 5-figure supplement 1-source data 3.tiff]

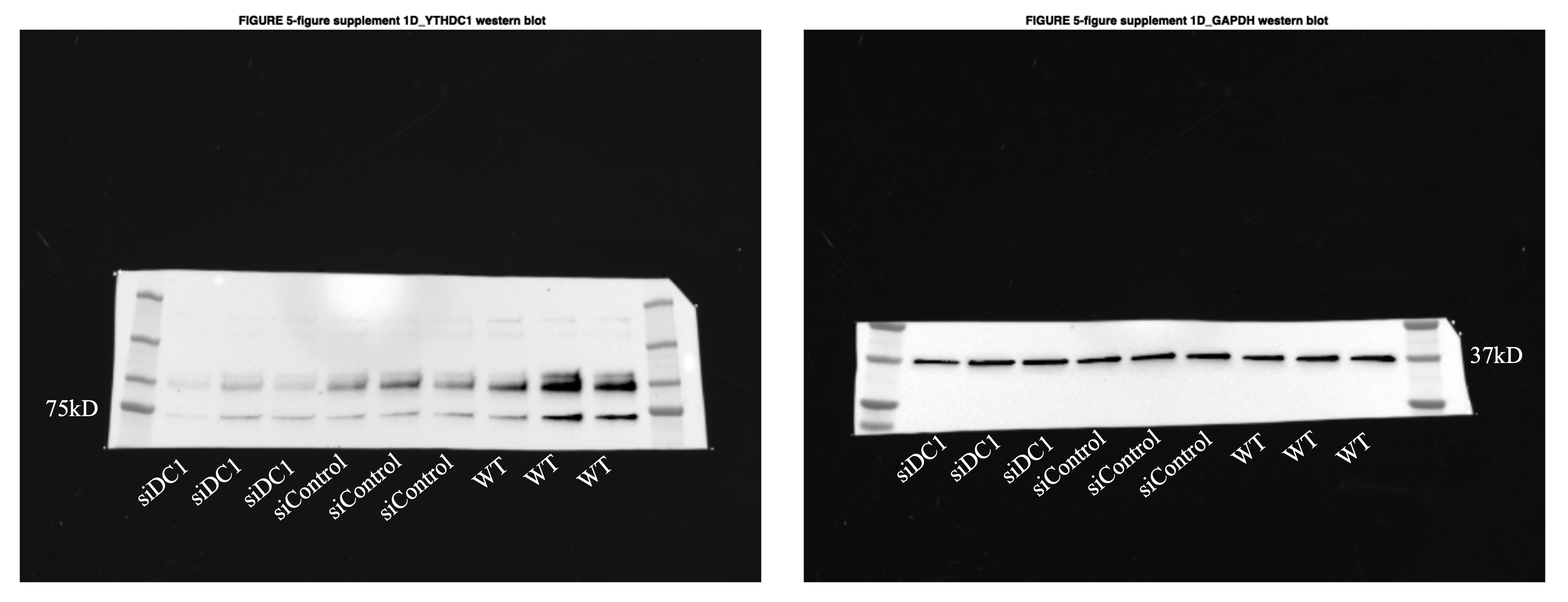

Supplement: Figure 5—figure supplement 1—source data 4. [file elife-85316-fig5-figsupp1-data4.zip › Figure 5-figure supplement 1-source data 4.tiff]

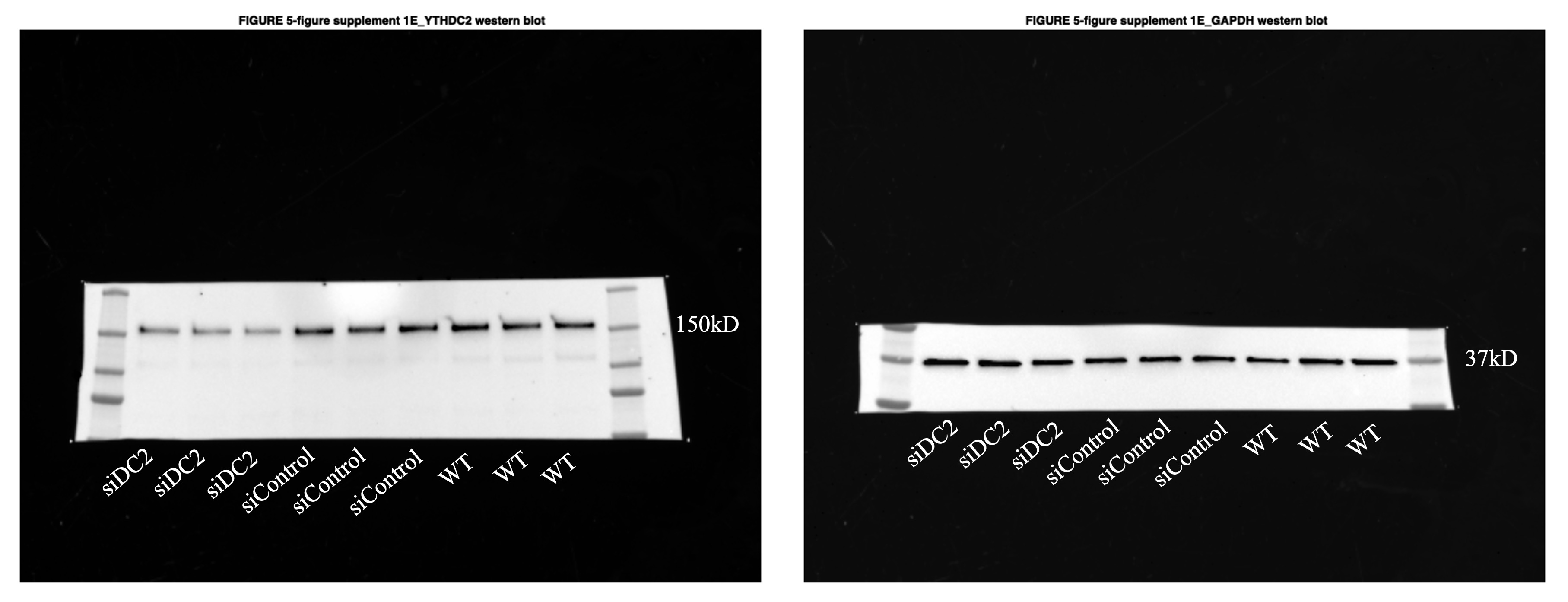

Supplement: Figure 5—figure supplement 1—source data 5. [file elife-85316-fig5-figsupp1-data5.zip › Figure 5-figure supplement 1-source data 5.tiff]
